# Gallic acid treats dust-induced NAFLD in rats by improving the liver’s anti-oxidant capacity and inhibiting ROS/NFκβ/TNFα inflammatory pathway

**DOI:** 10.22038/IJBMS.2021.51036.11603

**Published:** 2021-02

**Authors:** Hafseh Fanaei, Seyyed Ali Mard, Alireza Sarkaki, Gholamreza Goudarzi, Layasadat Khorsandi

**Affiliations:** 1 Physiology Research Center (PRC), Department of Physiology, School of Medicine, Ahvaz Jundishapur University of Medical Sciences, Ahvaz, Iran; 2 Persian Gulf’s Physiology Research Center (PRC), Department of Physiology, Ahvaz Jundishapur University of Medical Sciences, Ahvaz, Iran; 3 Air Pollution and Respiratory Diseases Research Center, Ahvaz Jundishapur University of Medical Sciences, Ahvaz, Iran; Environmental Technologies Research Center (ETRC), Ahvaz Jundishapur University of Medical Sciences, Ahvaz, Iran; 4 Cellular and Molecular Research Center, Department of Anatomical Science, School of Medicine, Ahvaz Jundishapur University of Medical Sciences, Ahvaz, Iran

**Keywords:** Dust, Gallic acid, NAFLD, Nrf2, Rat, TNF-α

## Abstract

**Objective(s):**

The burden of disease and death related to environmental pollution is becoming a major public health challenge, especially in developing countries. This study was designed to investigate the effect of dust exposure on liver function and its structure in rats. Gallic acid (GA) as a potent anti-oxidant was also used to treat NAFLD in rats exposed to dust.

**Materials and Methods:**

Twenty-four rats were randomly assigned into 3 groups: CA, Dust+N/S (after stopping dust exposure, rats received normal saline as vehicle, 1 ml, orally for 14 consecutive days), and Dust+GA (after stopping dust exposure, rats received GA at 100 mg/kg, orally for 14 consecutive days). Rats were exposed to CA/ dust for 6 weeks on alternate days. At the end of experiments, rats were anesthetized, their blood samples and liver sections were taken to perform molecular, biomedical and histopathological evaluations.

**Results:**

Dust exposure induced NAFLD features in rats. It increased the serum levels of liver enzymes, LDL, TG, cholesterol, MDA, and mRNA expression of NFκβ, TNFα, IL-6, HO1, and miRs [122 and 34a], while decreasing serum levels of HDL and liver TAC. Treatment with GA improved liver enzymes, serum levels of miRs, TG, expression of NFκβ, TNFα, IL-6, Nrf2, and HO1 and liver MDA and TAC levels, while it could not improve HDL, LDL, and cholesterol.

**Conclusion:**

This study showed dust exposure induced NAFLD in Wistar rats through inducing oxidative stress. Oxidative stress through activating the inflammatory pathways caused NAFLD features. Gallic acid treatment by inhibiting oxidative stress effectively protected liver function against dust induced inflammation.

## Introduction

Air pollution is a continuous challenge to public health that affects the general population. gaseous pollutants (NOx, ozone, sulfur dioxide, and carbon monoxide), persistent organic pollutants (pesticides, dioxins and furans), heavy metals (mercury, silver, lead, nickel, vanadium, manganese chromium, and cadmium), and particulate matters (PMs) constitute air pollution compounds(1).

Particulate matter (PM) is a generic term used for various kinds of air pollutants, with different size and composition produced by natural and anthropogenic phonemes (2). The size of the particle matters according to different categories have been defined as Ultrafine, Fine, and Coarse particles(aerodynamic diameter smaller than 0.1 µm, 1 µm and larger than 1 µm, respectively) (1).

 Most existing studies on air pollution have focused on particulate matters and gaseous pollutants (SO2, Ozone, and CO) and less on the effect of dust on the health status. Dust storms are natural phenomena in which soil particles are mass-transfered to another place, sometimes miles away from the point of dust storm origin (3). Climate changes, land drying, and desertification increase the probability of dust storm events(4). 

Dust storm carries fine and coarse particle matter fractions (5), bio-particulates and microorganisms, pollen, and related protein and lipid components (6). Despite high frequency of dust storms in the Middle East, few studies have focused on its public health effect (7).

Previous study has shown particle matter exposure via oxidative stress induced liver inflammation that has a critical role in liver pathogenesis (8). Hepatitis is a major contributing factor to the development of liver pathogenesis. Mice exposed to PM2.5 has been demonstrated to have increased mRNA expressions of inflammatory cytokines such as TNFα, IL-6 and exhibited Non-alcoholic fatty liver disease (NAFLD) (9). NAFLD is a spectrum of liver diseases ranging from simple non-alcoholic fatty liver (NAFL), to non-alcoholic steatohepatitis (NASH), and finally to liver irreversible cirrhosis (10).

Previous study has shown rats exposed to sub-chronic PM2.5 exhibited liver histopathological changes and elevated serum levels of AST and ALT (11). Exposure to ultrafine PMs has been indicated to increase hepatic levels of MDA which shows systemic oxidative stress and also enhances the gene expression of anti-oxidants related to Nrf2 (12).

Nrf2 as a transcription factor regulates anti-oxidant response and promotes cellular pathways protecting against oxidative stress. Nrf2 plays a vital role in various organs such as the brain, lung, and liver. Previous study has demonstrated that exposure to air pollutant components activated the Nrf2 pathway (13). 

Heme oxygenase 1(HO1) catalyzes degradation of heme, produces biliverdin and causes a general response to ROS induced oxidative stress (14). Pervious study has shown exposure to PM2.5 enhances liver inflammation and causes overexpression of mRNA HO1, TNFα, and IL-6 (11).

MicroRNAs (miRNAs) have an important role in physiological processes such as cell growth, differentiation, and development (15). miR-122 shows liver health status and represents as the most abundant a recognize specific miRs in liver (16). MiR-34a reflects liver damage, and a direct correlation between the serum level of miR-34 and liver injury has been proven (17).

Gallic acid (GA) as a phenolic compound, is abundant in vegetables, grapes, berries, tea, and wine (18-24). GA possesses different beneficial activities, including anti-inflammatory (24), anti-obesity (13) and hepatoprotective effects (25). This study was designed to evaluate the deleterious effects of dust exposure on liver function. Gallic acid as a potent anti-oxidant was used to treat NAFLD in rats exposed to dust.

## Materials and Methods

***Chemicals***

Gallic acid was purchased from sigma Aldrich Co. (Germany). Kits for measuring Malondialdehyde (MDA) and total anti-oxidant capacity (TAC) were purchased from ZellBio Co. (Germany). Kits for determination of ALT, AST, ALP, TG, cholesterol, and HDL were purchased from Pars Azmoon Company (Iran).

***Animals grouping***

Twenty-four adult male Wistar rats (weight 200–250 g) were purchased from the animal house of Ahvaz Jundishapur University of Medical Sciences. Animals were housed in a standard cage for 1 week before the experiment began. The animals were kept in the animal house of Ahvaz Jundishapur University of Medical Sciences, Ahvaz, Iran and were housed at a dark-light cycle of 12 hr and a temperature of 22±2 °C and had free access to standard rat chow diet and tap water.

**Figure 1:**
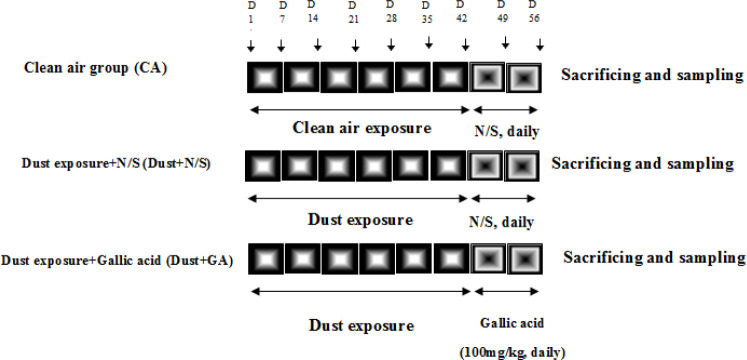
Experimental design of the rat model of non-alcoholic fatty liver disease by particulate matter (P.M)

The animals were divided randomly into 3 groups shown in Figure 1 (n=8 in each): CA (rats exposed to clean air), Dust+N/S (rats exposed to dust for 42 days on alternate days and then received normal saline as vehicle (1 ml/day, orally) for 14 consecutive days), and Dust+GA (animals exposed to dust for 42 days on alternate days and then received gallic acid at 100 mg/kg, orally for 14 consecutive days). All protocols and experiments were approved by the Experimental Animals Ethics Committee of Ahvaz Jundishapur University of Medical Sciences (IR.AJUMS.ABHC.REC.1397.060).

***Dust sampling and area of study***

Dust was collected in autumn (2018) from Ahvaz, the capital of Khuzestan Province, which is located in the southwest of Iran. Ahvaz is located at 31° 20 N, 48° 40 E geographically and has an 18 m elevation above sea level (26). To collect dust, we placed large dishes on the Golestan medical student’s dormitory roof for 3 months and then the settled down dust was collected and used in this study.

***Heavy metal analysis***

The dust sample was digested in an oven for 4 hr with a temperature of 170 °C. Before digestion, dust was exposed to a mixture of hydrofluoric, nitric, and hydrochloric acids in the Teflon bomb container. After 4 hr elapsed, the Teflon bomb was opened to remove evaporated acids. Distilled water and concentrated nitric acid were added after cooling. After shaking, the remaining liquid was filtered using a Whatman-42 paper. The obtained solution was then diluted and stored at 4 °C for analysis. Finally, it was analyzed for heavy metals using inductively coupled plasma optical emission spectroscopy (SPECTRO ICP-OES, Germany) (1). 

***Dust exposure***

**Figure 2:**
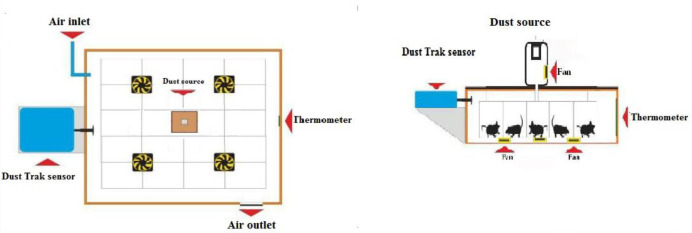
Schematic view of dust exposure cage used to induce non-alcoholic fatty liver disease (NAFLD)

To create a dusty environment, a Plexiglass cubic box (100 (length)×67 (width) ×26 (height) cm) with a 10 mm wall thickness was designed (Figure 2). A metal cage was made (93×63×17 cm) and divided into 20 equal square parts, located in a Plexiglass box. One rat was placed in each part of the metal cage. The distance between the bottom of the metal cage and the Plexiglas box floor was 7 cm. Five fans were placed in the center and each corner of the Plexiglass box to circulate the dust and provide an equal concentration of dust in all parts of the cage. In the left wall of the Plexiglass box a hole with a 2 cm diameter was created for sensor input. The sensor was connected to the DUST TRAK (TSI) device and the concentration of PM10 was recorded every second. In order to ventilate the air inside the box, an adjustable valve was fitted. Air pump (10 l/min) hoses entered the Plexiglass box through the right wall. Dust was in the plastic bottle. The upper part of the plastic bottle was made of pores. The hose connected to the adjustable peristaltic pump was inserted into the plastic bottle (containing dust). A plastic bottle was installed inside the plastic container inside which was a fan in (4×4 cm). The larger plastic container was fitted on the center of the Plexiglass box door. A hole was created on the center box door to allow the dust. Whole body exposure was performed 5 hr per day, 3 days per week (6 weeks) on alternate days for 90 hr combined. The concentration of PM10 during the study was 500–2000 µg/m^3^.

On the 56^th^ day of the experiment and before sacrificing, animals were fasted overnight. All rats were anesthetized by ketamine and xylazine (80+6 mg/kg, IP, respectively), after inducing deep anesthesia, the abdominal cavity was opened with a longitudinal incision. Blood collection was performed via cardiac puncture, and two samples of liver tissue were rapidly removed, frozen in liquid nitrogen and then stored at -80 °C until performing molecular and enzymatic analysis (27). Another liver tissue sample was also removed and fixed in formalin solution (10%).

***Serum biochemistry analysis***

Collected blood samples were centrifuged (6000 g for 10 min) and stored at -80 °C. The serum levels of aspartate aminotransferase (AST), alanine aminotransferase (ALT), alkaline phosphatase (ALP), and the level of total cholesterol, triglyceride (TG), and high-density lipoprotein (HDL) were determined using an automatic serum Automatic analyzer (BT 1500-A-A, Rome, Italy) (28). Low-density lipoprotein-cholesterol (LDL-C) was calculated as total cholesterol – (HDL-C + triglyceride /5).

***Histopathological analysis of liver***

Formaldehyde fixed liver tissue was embedded in paraffin and sectioned (5 µm) by using a microtome. The sections were stained by hematoxylin and eosin. Histopathological analysis was done blindly under a light microscope.

***Liver histology scoring***

The following grading method was used to determine the histopathological changes of the liver. The fatty change was graded according to the percentage of hepatocytes containing macrovesicular fat (grade 1: 0–25%; grade 2: 26–50%; grade 3: 51–75%; grade 4: 76–100%) (29). The degree of inflammation and accumulation of RBCs was expressed as the mean of 10 different fields within each slide that had been classified on a scale of 0–3 (0: normal; 1: mild; 2: moderate; 3: severe) (30).

***Assessment of the activity of anti-oxidants and level of lipid peroxidation***

The frozen liver tissue was homogenized in 1 ml PBS (pH7.4) and then centrifuged (15000 rpm for 15 min). The total anti-oxidant, TAC and MDA levels, in homogenates of liver tissue were measured using specific kits according to the manufacturer’s instructions.

***Measurement of the expression of miRs and mRNAs***

Total RNA (miRNAs and mRNAs) were extracted from the frozen serum samples using miRNeasy/Plasma kit and RNeasy plus mini kit, respectively (Qiagen, GmbH, Germany). After determination of the purity and concentration of extracted RNA, cDNA was synthesized (Qiagen, GmbH, Germany). To quantify the expression levels of studied miRNAs (122 and 34a) and mRNAs (NFκB, TNFα, IL-6, Nrf2 and HO1), semi-quantitative real-time PCR (qRT-PCR) was performed. 

***Statistical analysis***

Results are expressed as means±standard error of means (SEM). One-way analysis of variance (ANOVA) with LSD *post hoc* tests were used for the identification of significant differences for multiple comparisons among the studied groups (IBM SPSS statistics ver. 16). 

## Results

***The origin of the collected dust***

**Figure 3:**
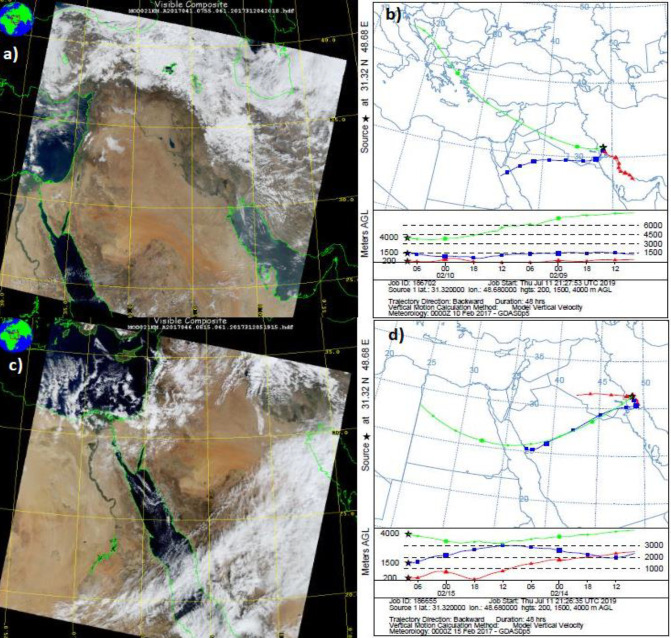
a) Backward HYSPLIT trajectory of the dust in Ahvaz on October 26, 2018, b) associated MODIS image over the region

One of several dust events that occurred in autumn 2018 is demonstrated based on the Hybrid Single Particle Lagrangian Integrated Trajectory (HYSPLIT) model and MODIS data in Figure 3. The highest PM10 level of this event recorded 725 µg/m^3^ at 05:00 AM on October 26^th^, 2018, though there were other dust storms in the same period which reduced visibility in Ahvaz with more PM10 severity. PM10 concentration was greater than 2000 µg/m^3^. It appears that Saudi Arabia was the main source of the dust storm. 

***Analysis of the given dust***

Analyzing the composition of the given dust for determining the heavy metal content showed the existence of 25 heavy metals: Ag, Al, As, B, Ba, Be, Cd, Co, Cr, Cu, Hg, Li, Mn, Mo, Ni, Pb, Sb, Se, Si, Sn, Sr, Ti, V, and Zn. The concentrations of Aluminum, Manganese, and Zinc in the given dust were the highest compared with the blank sample. 

***Changes in Macroscopic appearance and microscopic***

***architecture of the liver following exposure to dust***

To elucidate *in vivo* effects of whole body exposure to dust, male Wistar rats under normal diet were exposed to dust or clean air (CA) for 42 days. We tried to create a similar situation to real life by this way of exposure. During the exposure time period, the mean concentrations of PM10 and PM2.5 in the exposure chamber were 1493 and 448.3 µg/m^3^ and it was 50 µg/m^3^ for the CA group.

As shown in Figure 5B, inflammation, accumulation of RBCs, and fatty deposit in the liver were seen in the Dust+N/S group. No sign of fatty deposit was observed in the GA treatment group but mild inflammation and accumulation of RBCs were seen in this group. H&E staining revealed that inflammation, accumulation of RBCs, and fatty deposit in Dust+N/S rats were significantly higher than in the clean air group while GA treatment significantly reduced these levels (Table 2).

***Dust exposure increased serum levels of miR-122 and miR-34a while gallic acid treatment decreased these levels***

As demonstrated in Figure 6 (a and b), the serum levels of miR-122 and miR-34a remained up 14 days after stopping dust exposure (*P*˂0.05 in both). Gallic acid treatment (100 mg/kg, 14 consecutive days) significantly decreased the levels of mir-34a and miR-122 compared with Dust+N/S group.

***Gallic acid increased total anti-oxidant capacity (TAC) while it decreased hepatic malondialdehyde (MDA) levels in dust exposed rats***

As indicated in Figure 7, dust exposure significantly decreased hepatic TAC levels in Dust+N/S rats compared with the clean air group while it increased the hepatic MDA level (*P*˂0.05 in both). Treatment with GA at 100 mg/kg reverted TAC and MDA levels to normal.

***Gallic acid improved liver enzyme disturbances in rats exposed to dust***

As demonstrated in Figure 8, the dust exposure effect continued after stopping the dust exposure and resulted in significant elevation in serum levels of AST, ALT, and ALP in comparison with the CA group (Figures 8 a-c). Gallic acid treatment improved these levels. 

***Gallic acid improved lipid profile in rats exposed to dust***

Rats exposed to dust for 6 weeks and then received N/S (IP, 1 ml, 14 consecutive days) exhibited significantly increased serum levels of TG, cholesterol, and LDL compared with clean air group while showing decreased HDL serum levels (Figures 9a-d). GA treatment (at 100 mg/kg for 14 consecutive days) after stopping dust exposure significantly prevented any increase in serum levels of TG. But could not significantly improve serum levels of HDL, LDL, and cholesterol. 

***Effect of dust exposure and gallic acid treatment on mRNA expression of NFκβ, pro- inflammatory cytokines, Nrf2 and HO1***

As shown in Figure 10 (a-c), exposure to dust increased the mRNA expression levels of TNFα, NFκβ, and IL-6 in Dust+N/S rats compared with the CA group (*P*<0.05). GA treatment after stopping dust exposure protocol significantly decreased these levels (*P*˂0.05 in all cases). Nrf2 mRNA expression in rats exposed to dust increased significantly compared with the clean air group (*P*<0.05). This increase in rats treated with GA was much more than in rats exposed to dust alone (*P*<0.01). Expression of HO1 mRNA in Dust+N/S group which was exposed to dust for 6 weeks and then received 1 ml N/S (IP, 14 consecutive days) increased but not significantly compared with rats exposed to clean air. GA treatment significantly increased these levels compared with rats exposed to clean air (*P*<0.05). 

## Discussion

In the present study, we determined the effect of dust whole body exposure on rat liver function and structure. This study showed that sub-chronic exposure to dust led to induction of NAFLD in rats through increasing the production of liver MDA, decreasing liver total anti-oxidant capacity, disruption of lipid profiles and liver enzymes, increasing the level of anti and pro-inflammatory cytokines (HO1, IL-6, and TNFα), changing the levels of nuclear transcription factors (NF-κβ and Nrf2), and elevating the serum levels of miRNAs (miR-122 and 34a). Gallic acid treatment improved liver function against dust harmful effects through improving almost all the studied variables. 

Dust composition is dependent on dust origin. There is a direct correlation between dust composition and its side effects. Prior studies have shown dust components (PMs or heavy metals) when evaluated individually could endanger health status (31-34). Human studies have shown that there is direct correlation between the serum levels of heavy metals (cadmium, cobalt, chromium, nickel, lead, zinc, and aluminum) and development of NAFLD features (33).Our results showed that heavy metal concentration in the given dust were higher than normal levels. Higher concentration of heavy metals such as Al, As, Cd, and Cu in the given dust compared with the sample blank could be an important reason for functional and morphological changes of liver tissue in rats exposed to dust.

Present results showed that whole body dust exposure induced NAFLD features and NAFLD signs remained 2 weeks after stopping dust exposure. Our microscopic evaluations demonstrated dust exposure represented inflammation, accumulation of RBCs, and fatty deposit in rat livers compared with the CA group. In the livers of GA treated rats no signs of fatty deposit were seen while inflammation and accumulation of RBCs were obvious in this group. Therefore, it seems that the studied GA dose or treatment time probably were not enough to completely treat dust-induced liver injuries. However, this dose was selected according to previous reports that have shown GA at 100 mg/kg is effective in liver disorders (35).

Earlier studies have shown heavy metals are cable of oxidative stress induction (36). Given dust analysis showed that the amount of heavy metals was dramatically high. Thus, these findings show that oxidative stress induction occurred due to the high concentration of heavy metals along with other components in the given dust. However, increased level of MDA (as a marker of lipid peroxidation) related to dust exposure could represent oxidative stress. The prior studies have also reported that UFP exposure via systemic oxidative stress induction increase MDA levels (12). In agreement, the present study also showed that liver MDA levels increased in dust exposed rats compared with CA. These findings concluded that dust exposure induced liver oxidative stress, increased lipid peroxidation, damaged hepatocyte cell, and increased cell membrane permeability which leads to an increment in serum levels of ALT, AST, ALP, and miR-122. This study showed that liver MDA levels in the Dust+GA group were significantly lower than in Dust+N/S rats. Previous reports have indicated the ROS scavenging activity and anti-oxidant promoting activity of gallic acid in the liver on injuries CCl_4_-hepatotoxicity (37, 38). Therefore, it can be concluded gallic acid is able to treat the harmful effects of dust induced oxidative stress. 

Liver structural damage and cellular leakage lead to over release of liver amino transaminases and ALP from hepatocytes and elevated these levels in serum (39). The present results showed after stopping dust exposure (14^th^ day), serum levels of AST, ALT, ALP, miR34a, and miR122 in Dust+N/S rats were significantly higher than in the CA group. It showed that the harmful effects of dust continue even after stopping dust exposure. Improving these levels in GA treated rats shows that dust-induced liver injuries critically need therapeutic actions. 

Our study showed that levels of TG, cholesterol, and LDL in the Dust+N/S group were significantly higher while the HDL levels were significantly lower than in the CA group. Previous studies have shown air pollution exposure increases adipose inflammation, insulin resistance, promote lipolysis, and elevates serum TG levels (40, 41). Therefore, the present outcomes were in agreement with previous studies that have shown that air pollution disrupts the lipid profile.

Gallic acid treatment could not improve serum levels of cholesterol, LDL and HDL while improving serum TG levels. Therefore, it seems dust exposure effect on lipid profile was severe and dose or treatment time probably were not enough to completely treat liver tissue injury against dust exposure injuries.

The current study showed that exposure to dust and GA treatment increased the gene expressions of Nrf2 and HO1 in liver tissue compared with the clean air group. Previous study has shown airborne particulate matter increases Nrf2 and HO1 mRNA expression (12) .The higher level of Nrf2 expression in rats treated with gallic acid (Dust+GA) can be due to additive effect of two Nrf2 inducers, dust and gallic acid. Therefore, the present outcomes were in agreement with previous reports that have shown that dust increased mRNA expression of Nrf2 (significantly) and HO1(no significantly), while gallic acid pretreatment had much more effect on Nrf2 and HO1 expression (42).

Oxidative stress activates the NFκβ pathway and increases the expression of pro-inflammatory factors: TNFα, IL-1, and IL-6 (43). The present results showed that following dust exposure the mRNA levels of NFκβ, TNFα, and IL-6 increased significantly compared with the clean air group. Previous studies have shown exposure to PMs increased mRNA expression of NFκβ and inflammatory cytokines (9, 44). Therefore, taking these results together, it can be concluded that exposure to dust as shown here, or its components (PMs) as reported by previous studies, by activating the inflammatory pathways damaged the liver and induced hepatitis. The other results achieved in this study were significant decrease in mRNA levels of NFκβ and proinflammatory cytokines (TNFα, IL-1, and IL-6) in rats treated with GA. These findings were in agreement with previous reports (45).

Our finding showed that dust exposure caused a significant decrease in the liver TAC while gallic acid treatment improved this level. Air pollution components as oxidative stress stimulator disturb the oxidant-anti-oxidant balance and oxidant mediators conquered anti-oxidant defense. This situation makes organs susceptible to oxidative stress damages. Previous studies have shown that air pollution exposure reduces both serum and organ anti-oxidant levels (46). Another study also showed that gallic acid improves anti-oxidant capacity and exhibits protective effects on liver oxidative stress (47). Our findings were consistent with prior studies that have shown that dust exposure decreased anti-oxidant potency while gallic acid treatment increased TAC and improved anti-oxidant capacity.

**Table 1:** Lists the sequence of forward and reverse primers for amplifying each gene

Primers	Forward	Reverse
NFκβ	ACCCGAAACTCAACTTCTGT	TAACAGCTGGGGGAAAACT
TNFα	TGTGCCTCAGCCTCTTCTCATTC	CATTTGGGAACTTCTCCTCCTTG
IL-6	GGTCTTCTGGAGTTCCGTTT	AGTTGGGGTAGGAAGGACTA
Nrf2	CTCTCTGGAGACGGCCATGACT	CTGGGCTGGGGACAGTGGTAGT
HO1	TCAGCACTAGTTCATCCCAG	AAGCTTTCTTAGAGGCCCAA
GAPDH	TGCTGGTGCTGAGTATGTCGTG	CGGAGATGATGACCCTTTTGG

No-template negative control (H_2_O) was routinely run in every PCR. The levels of miRNAs and mRNAs expression were respectively normalized with RNU6 (as an internal control) and glyceraldehyde-3-phosphate dehydrogenase (GAPDH) and the fold change was calculated using the 2^–ΔΔCt^ formula

**Figure 4:**
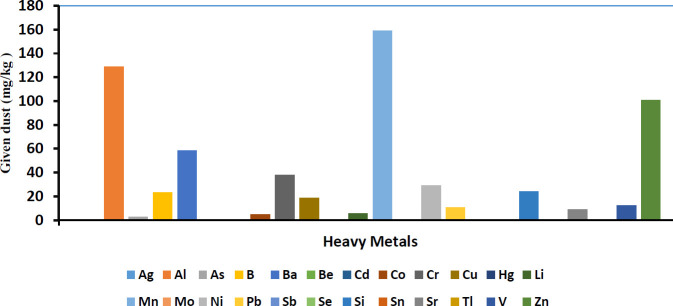
The overall mean content of the investigated heavy metals in collected dust

**Figure 5:**
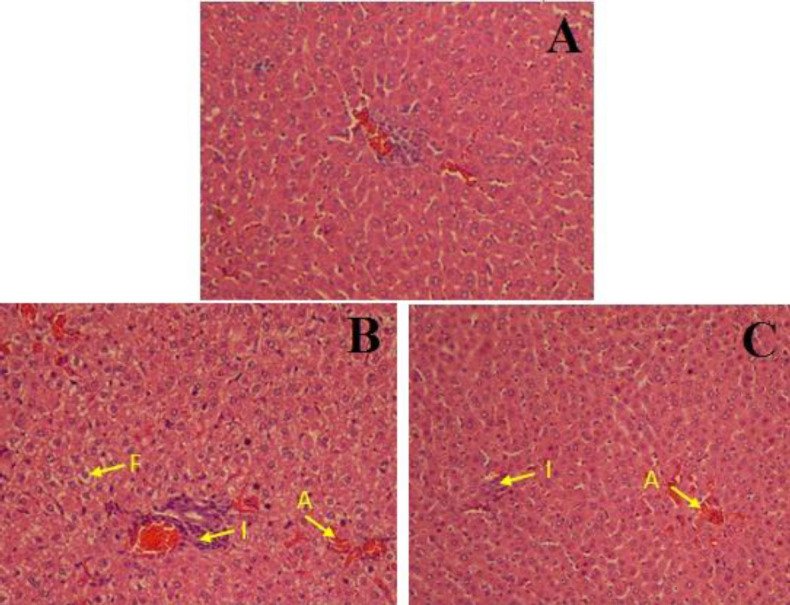
Representative microscopic images (magnification ×250) of H&E stained liver sections following dust and clean air (CA) exposure. (A) clean air group showed normal appearance and there were no histopathological changes, (B) liver in Dust+N/S rats showed inflammation (I), accumulation of RBCs (A), macrovesicular fatty deposit (F) and (C): gallic acid treatment group showed mild inflammation and red blood cell accumulation but no signs of fatty deposit

**Table 2:** NAFLD grading and staging in Wistar rats exposed to clean or dusty air

Fatty deposit	RBCs accumulation	inflammation	Groups
.000± 0.00	0.02±0.00	0.03±0.00	CA
0.52± 0.83*	1.27±0.31 *	1.1±0.28*	Dust+N/S
.000± 0.00	0.74±0.00 *#	0.53±0.11 *$	Dust+GA

*P<0.05 compared with CA group. $*P*<0.05 compared with Dust+N/S group

**Figure 6:**
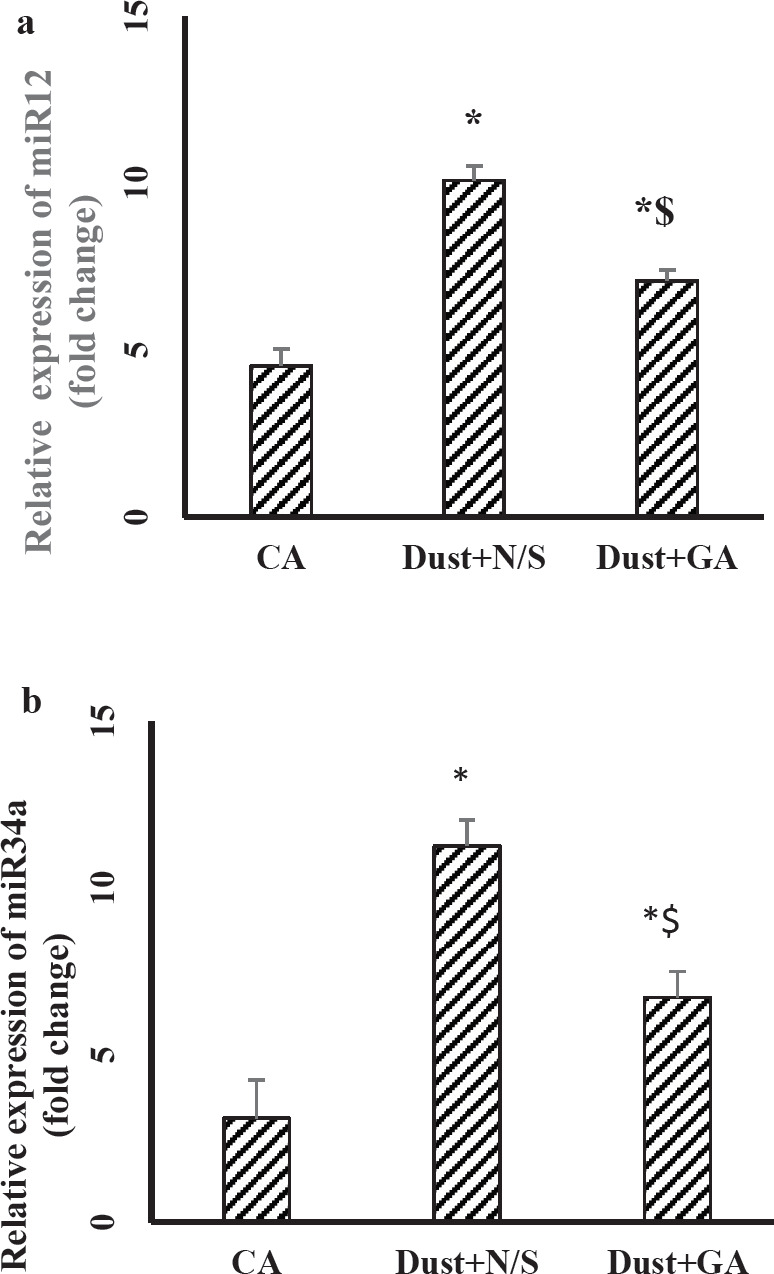
Effect of dust exposure and Gallic acid treatment on serum levels of miR-122 (a), miR-34a (b). The data are presented as the mean ±SEM. *P˂0.05 compared with CA group; and $ *P*˂0.05 compared with Dust+N/S group

**Figure 7:**
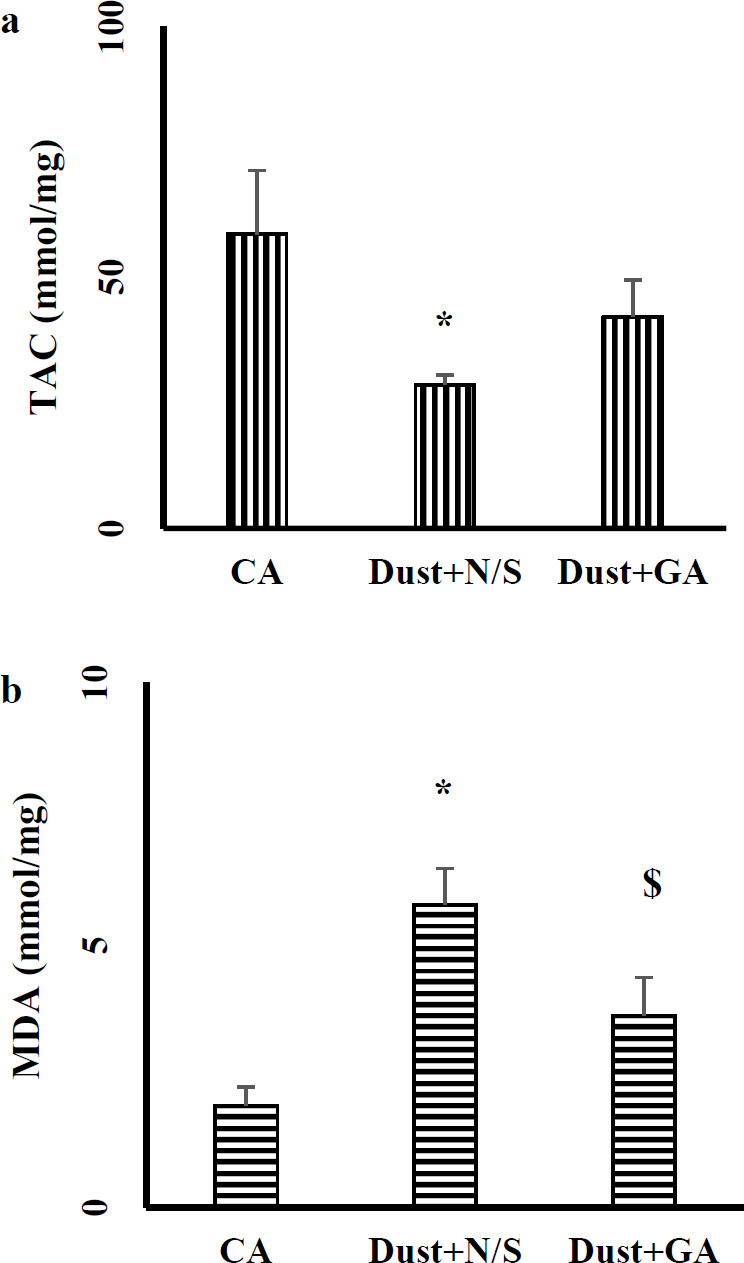
Gallic acid decreased hepatic MDA levels while increasing the hepatic level of TAC in rats exposed to dust. Data are expressed as means±SEM.*P<0.05 compared with CA group and $ *P*<0.05 compared with Dust+ N/S

**Figure 8:**
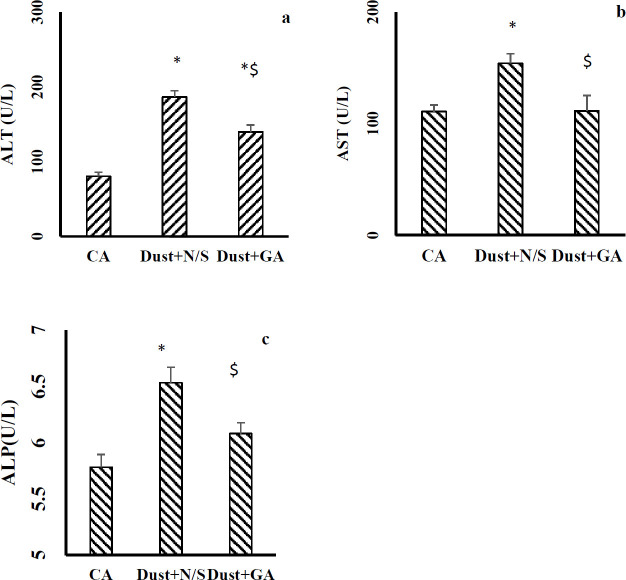
Gallic acid improved liver enzyme disturbances in rats exposed to dust. (a) Serum level of ALT; (b) Serum level of AST; and (c) Serum level of ALP. Data are presented as the mean±SEM. *P<0.05 compared with clean air group; $ *P*<0.05 compared with Dust+N/S group

**Figure 9:**
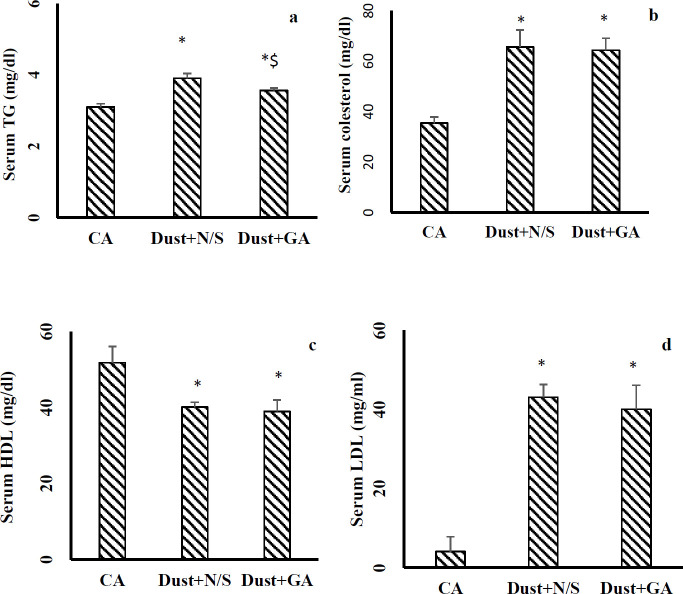
Dust exposure effect on lipid profile continued after stopping dust exposure. (a) Serum levels of triglyceride; (b) cholesterol; (c) HDL; and (d) LDL. The data are presented as the mean±SEM. *P<0.05 compared with clean air group; $* P*<0.05 compared with Dust+N/S group

**Figure 10:**
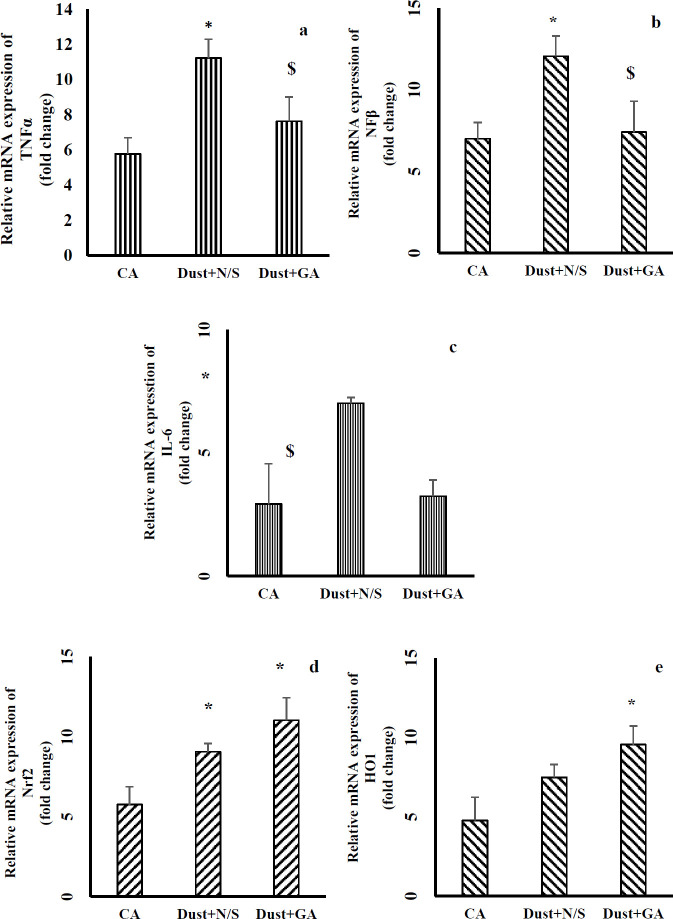
Effect of dust exposure and gallic acid treatment on mRNA expression of NFκβ, pro- inflammatory cytokines, Nrf2, and HO1

*P<0.05 compared with clean air group; $P *P*<0.05 compared with Dust+N/S. TNFα: tumor necrosis factor, IL-6: interleukin 6, NF-κβ: nuclear factor κβ, HO1: heme oxygenase-1, Nrf2: nuclear factor erythroid related factor 2

## Conclusion

This study showed that exposure to dust induced NAFLD in rats through induction of oxidative stress which in turn increased liver MDA levels. Oxidative stress through activating the inflammatory pathways caused NAFLD features including fatty deposit, inflammation, and RBCs accumulation. Gallic acid treatment improved liver structure and function against dust induced inflammation by inhibiting oxidative stress. 
